# The Fluidic Shear Stress Loading Method Enables Mechanobiological Stimulation in an On-Chip Pump-Integrated Microphysiological System

**DOI:** 10.3390/mi16091051

**Published:** 2025-09-15

**Authors:** Jin Hong Yap, Satoshi Ishizaki, Hiroko Nakamura, Kenta Shinha, Hiroshi Kimura

**Affiliations:** 1 Department of Mechanical Engineering, School of Engineering, Tokai University, 4-1-1 Kitakaname, Hiratsuka 259-1292, Japan; 2 Micro/Nano Technology Center, Tokai University, 4-1-1 Kitakaname, Hiratsuka 259-1292, Japan

**Keywords:** microphysiological system (MPS), organ-on-a-chip, BioStellar™ plate, fluidic shear stress (FSS)

## Abstract

Microphysiological systems (MPSs), such as organ-on-a-chip platforms, are promising alternatives to animal testing for drug development and physiological research. The BioStellar™ Plate is a commercial MPS platform featuring an open-top culture chamber design with on-chip stirrer pumps that circulate culture medium through six independent, dual microchannel-connected chamber multiorgan units. Although this design enables a circular flow, the open-top culture chamber format prevents the application of fluidic shear stress, a force that cells experience in vivo, which affects their behavior and function. To address this, we developed two fluidic shear stress attachments for the BioStellar™ Plate. These attachment channel fluids provide controlled mechanical stimulation to cultured cells. The flow dynamics were simulated using COMSOL Multiphysics to estimate shear stress levels. The attachments were fabricated and validated through fluorescent bead tracking and biological assays. The FSSA-D is designed for flat-bottom standard cell cultures, while the FSSA-I is designed for epithelial monolayers, enabling the application of fluidic shear stress across the basal membrane. Experiments with intestinal epithelial cells (Caco-2) demonstrated that both attachments enhanced cell barrier function under a fluidic environment, as indicated by higher transepithelial electrical resistance (TEER). These findings demonstrate that the attachments are practical tools for mechanobiology research with MPS platforms.

## 1. Introduction

In drug development, typical experiments for drug discovery often use animal experiments and cell-based assay tests. Animal experiments have various issues, such as ethical considerations and species differences. Cell-based assays have issues with cultured cells that do not retain the organ’s function in vitro. In recent years, in medicine and life sciences, microphysiological systems (MPSs), including organ-on-a-chip (OoC), are attracting attention as promising technologies that can reproduce in vivo-like tissue structures and fluid flow on microfabricated platforms. MPSs are increasingly being utilized to evaluate drug pharmacokinetics, toxicity, absorption, metabolism, and interorgan interactions under controlled in vitro conditions [1,2,3,4]. Multiorgan microphysiological systems (MO-MPSs) enable the co-culture of multiple organ-derived cells, allowing the study of complex organ–organ interactions in real time and contributing to more accurate drug evaluation systems [5,6,7,8].

A key element for reproducing physiological conditions in MPSs is the application of dynamic fluid flow to simulate blood circulation. Conventionally, syringe and peristaltic pumps have been used to generate perfusion flow; however, these external pump systems require complex tubing connections, present contamination risks and air bubbles, and consume large volumes of culture medium, increasing experimental costs and reducing throughput [9,10]. Various integrated pumping methods, including pneumatic pumps, membrane pumps, and gravity-based systems, have been developed to address these issues. However, many still involve complicated operations or closed-channel configurations that reduce usability for long-term culture and co-culture setups.

To overcome these limitations, a stirrer-based kinetic pump system was previously developed, which generates fluid circulation by rotating miniature magnetic stir bars embedded inside the culture device [11]. This technology led to the development of the Kinetic Pump Integrated Microfluidic Plate (KIM-Plate), which combines the stirrer-based kinetic pump with an open-top culture chamber multiorgan microphysiological design [12]. The KIM-Plate comprises six independent MO-MPS units conforming to ANSI/SBS microplate standards, allowing direct use with commercially available cell culture inserts and disks. Open-top culture chambers of the KIM-plate simplify handling and enable the co-culture of multiple organ models. However, the KIM-Plate was made of black polymer, which made it difficult to check the stirrer rotation. Sumitomo Bakelite developed the BioStellar™ Plate as a redesigned next-generation system based on the KIM-Plate concept to improve usability. The design principles of the BioStellar™ Plate’s open-top culture chamber architecture and kinetic pump integration provide a versatile framework that can be adapted for use with other commercial MPS platforms. This approach offers simplified handling, reduced contamination risk, and increased experimental throughput across various MPS platforms. The BioStellar™ Plate retains the exact kinetic pump mechanism and open-top culture chamber design but employs transparent polystyrene for full compatibility with standard microscopy. While the BioStellar™ Plate provides continuous perfusion, it cannot apply controlled fluidic shear stress (FSS), which is an important factor influencing cellular morphology, differentiation, and barrier function under physiological conditions, because of the open-top culture chambers.

To address this limitation, we developed FSS loading attachments capable of guiding fluid flow and introducing controlled FSS onto epithelial cells cultured within the BioStellar™ Plate in this study. We designed and fabricated fluidic shear stress attachments (FSSAs) for the BioStellar™ Plate, conducted flow simulations, and verified their hydrodynamic behavior through experiments. We successfully introduced controlled FSS during dynamic perfusion by applying these attachments to epithelial cell cultures. The controlled FSS environment created by these attachments enables the investigation of cellular responses across multiple cell types, including endothelial and epithelial cells, as well as potentially other mechanosensitive cell populations that are crucial for modeling various organ systems and disease states. As a result, cells exposed to FSS showed improved growth and organization compared to static cultures, demonstrating the effectiveness of this approach in enhancing the BioStellar™ Plate for future microphysiological applications. The modular design approach of these microfluidic accessories will adapt to other commercial MPS platforms, potentially expanding the utility of controlled shear stress applications across the broader MPS research community.

## 2. Materials and Methods

### 2.1. BioStellar™ Plate

**Figure 1:**
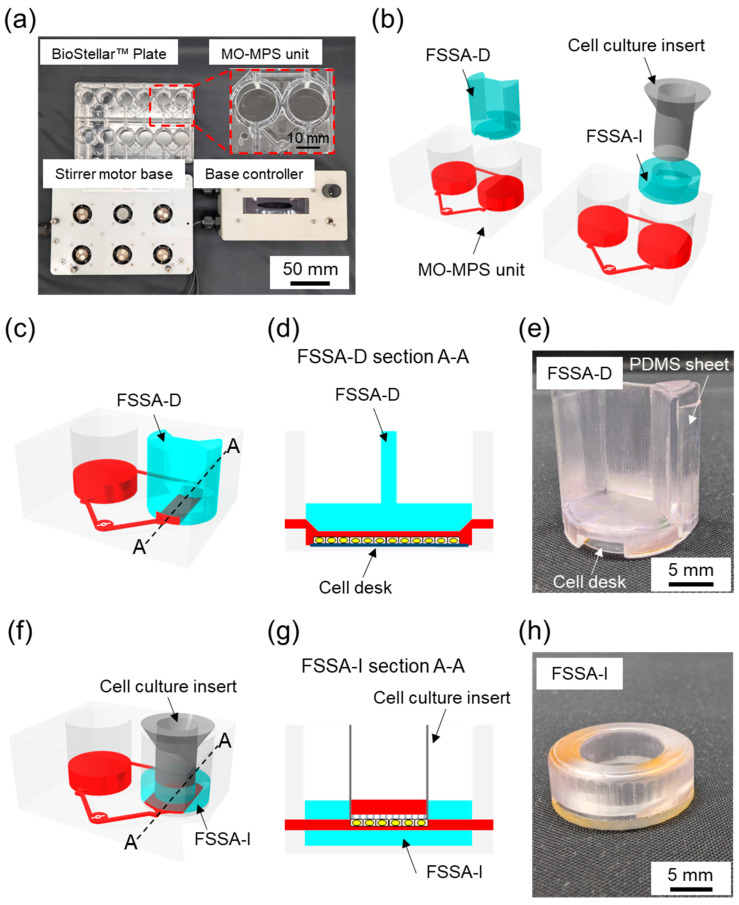
BioStellar™ Plate system and the design of the fluidic shear stress attachments (FSSAs). (**a**) Photograph of the BioStellar™ plate system, including the BioStellar™ Plate with six independent multiorgan culture units, a stirrer motor base for kinetic flow generation, and a stirrer motor base controller for adjusting rotation speeds. (**b**) A 3D illustration of the fluidic shear stress attachment—cell desk (FSSA-D) and the insert-based fluidic shear stress attachment cell culture insert (FSSA-I) with the BioStellar™ Plate. (**c**) A 3D schematic showing the FSSA-D mounted within a BioStellar™ Plate unit, demonstrating how the attachment integrates with the chamber layout to channel fluid flow directly over the cell desk surface. (**d**) Cross-sectional diagram of the FSSA-D configuration, highlighting the linear flow path created across the flat culture surface for uniform fluidic shear stress application. (**e**) Fabricated FSSA-D, designed to support flat-bottom culture with the cell desk. (**f**) A 3D schematic showing the FSSA-I assembled within a BioStellar™ Plate unit, illustrating how the FSSA positions flow beneath the inserted membrane for basal-side stimulation. (**g**) Cross-sectional diagram of the FSSA-I configuration, depicting the flow pathway directed along the underside of a cell culture insert to deliver controlled fluidic shear stress. (**h**) Fabricated FSSA-I, designed to accommodate standard cell culture inserts.

The BioStellar™ Plate (Sumitomo Bakelite, Tokyo, Japan) consists of six independent MO-MPS units that conform to ANSI/SBS microplate standards (128 mm × 85 mm × 40 mm). Each unit features dual microchannel-connected open-top culture chambers with the same diameter and configuration as standard 24-well plates, making it fully compatible with conventional cell culture protocols. Commercially available cell culture inserts and disks can be used directly within the plate without any modifications (Figure 1a). The entire plate body was fabricated from optically clear polystyrene (PS) using injection molding. This enhances imaging compatibility and allows for seamless observation during culture. The microfluidic architecture connects each chamber pair by microchannels (1.0 mm wide × 0.3 mm high). These channels were positioned 1.2 mm above the bottom of the chamber. This construction limited direct fluid shear stress on cells.

Culture medium circulation was achieved through an embedded stirrer-based kinetic pump driven by a 3 mm × 0.2 mm stainless-steel stirrer bar. The clockwise rotation of a magnetic rotor beneath the plate generates forward flow through the main channel. In contrast, backflow through the secondary channel completes the loop, creating a continuous circulation system. The BioStellar™ Plate was designed to be used with a custom-built magnetic stirrer motor base that holds six magnetic rotor units, each driven by DC axial fans (108-AFB0412HHA-TA5F, Delta Electronics, Taipei, Taiwan). The base and its controller, produced by Microfluidic System Works, allow simultaneous control of rotor speeds, ensuring consistent and adjustable flow across all six MO-MPS units (Figure 1a). The system could reach rotation speeds of up to 6500 rpm in its current configuration, allowing for a broad operational range that generates various flow rates suitable for both low-shear and high-shear experimental conditions.

### 2.2. Fluidic Shear Stress Attachments

To specifically introduce regulated FSS to cell cultures within the BioStellar™ Plate, we developed two customized fluidic shear stress attachments (FSSAs), each designed to accommodate different cell culture formats while maintaining compatibility with the BioStellar™ Plate (Figure 1b).

The first attachment, the Fluidic Shear Stress Attachment—Cell Desk (FSSA-D), supports flat-surface cultures on the cell desk (Figure 1c,d and Figure S1a). Its primary function is to establish a controlled flow channel directly above the cultured cells. The centrally aligned straight microchannel measures 5.5 mm in width and 0.4 mm in height after cell desk installation. To maintain consistent flow, the channel incorporates 35° slopes at both the inlet and outlet, which guide fluid into the central region. The FSSA-D body is 15.9 mm in diameter and 17.0 mm in height, with a concave slot at the bottom (13.7 mm diameter; 0.3 mm depth) to secure the cell desk (MS-92132, Sumitomo Bakelite, Tokyo, Japan). Two grooves, each 0.5 mm deep and 7.6 mm wide, run along the sidewalls to accommodate PDMS sheets and form a watertight seal with the BioStellar™ Plate. This channel design enables unidirectional laminar flow across the apical cell surface, closely replicating physiological fluidic shear stimulation observed in blood vessels and other flow-exposed tissues (Figure 1e).

The second attachment, the Fluidic Shear Stress Attachment—Insert (FSSA-I), is engineered for cell culture inserts compatible with 24-well plate formats (Figure 1f,g and Figure S1b). This design applies fluidic shear stress to the basal surface of cell monolayers grown on microporous membranes. The FSSA-I consists of a circular body (16.0 mm diameter; 5.5 mm height) with a central insertion port (4.0 mm height; 9.4 mm diameter) that securely holds the insert. The flow-guiding region beneath the insert is a rectangular channel (9.3 mm width; 0.5 mm height), which ensures even perfusion of medium along the basal membrane surface. The bottom structure, made from 1 mm thick acrylic, provides rigidity and optical clarity for microscopy. By directing flow beneath the membrane, the FSSA-I enables localized fluidic shear stress on the basal side of epithelial layers, replicating dynamic microenvironments found in intestinal, renal, or vascular systems (Figure 1h). Both attachments utilize the BioStellar™ Plate’s circulation system to generate physiologically relevant mechanical stimulation on cultured cells, eliminating the need for external pumps or complex tubing.

### 2.3. Fabrication of the FSSAs

FSSAs were fabricated using a high-resolution 3D printer Formlabs Form 3B+ (PKG-F3B-COMPLETE-EW-1, Formlabs, MA, USA) with BioMed Clear resin (RS-F2-BMCL-01, Formlabs, MA, USA), a biocompatible photopolymer suitable for cell culture applications. The FSSAs’ geometries were designed using AutoCAD 2019 (Autodesk, CA, USA) and exported as STL files, which were subsequently processed in PreForm software (Formlabs, MA, USA). The models were printed using default settings with a layer thickness of 50 µm to achieve high-dimensional precision and smooth surface quality suitable for microfluidic use. Following printing, the attachments were washed in isopropyl alcohol (IPA) using the Form Wash (FH-WA-02, Formlabs, MA, USA) for 15 min to remove uncured resin from channel structures and surfaces. The printed parts were then post-cured using Form Cure (FH-CU-01, Formlabs, MA, USA) at 60 °C for 30 min, ensuring complete crosslinking and the mechanical stability of the material. The cured attachments underwent a detailed multi-step solvent-washing protocol designed to remove toxins from the printed material and prepare it for further biological use. Initially, each FSSA was submerged in acetone and cleaned with an ultrasonic washer for 60 min. After the acetone wash, the attachments were rinsed in IPA for 30 min using an ultrasonic washer to ensure that any traces of acetone were thoroughly removed. The attachments underwent three consecutive washes, each lasting 30 min, with ion-exchanged water, again using an ultrasonic washer. Finally, the attachments were sterilized by autoclaving at 120 °C for 20 min. This high-temperature process eliminated any potential microbial contamination. After autoclaving, the FSSAs were allowed to cool overnight to minimize the risk of cracking caused by thermal stress, ensuring they were safe and ready for further applications.

The bottom of FSSA-D was installed using the cell desk and double-sided tape, forming a connected perfusion chamber. To reinforce the lateral fit and maintain structural alignment within the BioStellar™ Plate, a 0.5 mm thick PDMS sheet was applied along the sidewall’s grooves of the attachment (Figure 1e). Conversely, the FSSA-I was assembled onto a custom-fabricated acrylic base to accommodate a cell culture insert (Transwell^®^, 3450, Corning, New York, NY, USA) (Figure 1h). The components were joined using double-sided tape to ensure stable integration.

Following assembly, the FSSAs underwent gas sterilization to ensure sterility across all contact and interface surfaces. Before beginning biological experiments, the FSSAs were stored for at least one week to remove any potential cytotoxicity from residual sterilant gases.

### 2.4. Numerical Simulation in Flow

To evaluate the flow characteristics within the BioStellar™ Plate, 3D models of the cell culture chamber, FSSA-D, and FSSA-I were constructed using COMSOL Multiphysics 6.1 (COMSOL, Stockholm, Sweden) to assess the flow characteristics. The simulation chamber dimensions were chosen to replicate the actual geometry of the BioStellar™ Plate, rather than the standard 24-well plate dimensions. Standard 24-well plates typically feature wells with a diameter of approximately 15.6-16.5 mm and a total depth of 17.0 mm. The BioStellar™ Plate features open-top culture chambers optimized for the integrated kinetic pump system and operates under different parameters. The simulation domain consisted of a cylindrical chamber with a diameter of 16.4 mm and a height of 4.7 mm, with two fluidic channels positioned at opposite ends. The chamber diameter of 16.4 mm matches the inner diameter of the BioStellar™ Plate culture chambers, which have a height of 4.7 mm, corresponding to the water level when filled with 1 mL of culture medium, the standard working volume for this system. Each channel was 1.0 mm wide and 0.5 mm high, serving as both the inlet and outlet, and representing the actual fluidic connections in the BioStellar™ Plate system. The working fluid was modeled using water properties at 37 °C, with minor adjustments to reflect physiological conditions. Simulations were performed at five different inlet flow velocities to analyze variations in velocity distribution across both FSSA designs.

### 2.5. Flow Observation

To experimentally validate flow simulations and characterize flow behavior, we performed particle tracking velocimetry using fluorescent microbeads (10 μm in diameter, 1.1 × 10^8^ particles/mL, 18142, Polyscience, IL, USA) as flow tracers. Instead of using the full BioStellar™ Plate, we employed a custom-fabricated PDMS-based open-top culture chamber model that resembled the BioStellar™ Plate architecture. The chamber model retained the essential features of the BioStellar™ Plate, including inlet and outlet water channels and a single cell culture chamber, but also included a glass bottom to facilitate high-resolution imaging (Figure S2).

The FSSAs were mounted onto the chamber model to replicate the in situ flow environment. Flow was driven by two syringe pumps programmed to mimic the pulsatile perfusion behavior generated by the BioStellar™ Plate. We recorded bead movement in the central region of each attachment using an Olympus IX71 (Olympus Corporation, Tokyo, Japan) inverted fluorescence microscope paired with a video camera (FDR-AX700, Sony, Tokyo, Japan) operating in super slow-motion mode (240 fps).

The captured videos were analyzed using FlowExpert64 Ver 1.3.0 software (Katokoken, Kanagawa, Japan), a particle image velocimetry (PIV) platform, to quantitatively extract flow velocity fields and estimate local FSS in the attachment channels. The Supplementary Information includes a representative video of the microbead flow behavior (Figure S3; Videos S1 and S2).

### 2.6. Estimation of FSS

FSS (*τ*) (Pa) was calculated from the velocity data using the following equation [13,14]:

$$\tau =\;\frac{6\mu }{h}ս$$

where *μ* is the viscosity of water at 37 °C (0.6913 mPa·s), *u* is the average flow velocity (m/s), and *h* is the channel height (m).

It is worth noting that the flow velocity obtained through microscopy reflects the peak velocity at the center (z-axis) of the microchannel rather than the average velocity across the entire cross-section, an important distinction when calculating FSS. Additionally, while FSS was calculated using standard SI units (Pascal, Pa) via the equation, the values reported throughout the results section are expressed in dyne per square centimeter (dyn/cm^2^) for consistency with the biological literature. For reference, 1 Pa is equivalent to 10 dyn/cm^2^. In cylindrical channels, the average velocity is generally assumed to be half the maximum. However, this relationship does not carry over to rectangular channels like the FSSAs. To address this, we used ANSYS Fluent 2022 R1 (Ansys, PA, USA) to simulate the precise channel geometries of both FSSA-D and FSSA-I and to determine the ratio between maximum and average velocities. From these simulations, correction factors of 1.55 for FSSA-D and 1.56 for FSSA-I were obtained and applied to convert the measured peak velocities into more accurate average velocities (Figure S4).

### 2.7. Cell Culture

Human epithelial colorectal adenocarcinoma cells (Caco-2, ATCC, Virginia, USA) were cultured in 100 mm culture dishes (3020-100, Iwaki, Shizuoka, Japan) using Dulbecco’s Modified Eagle Medium (DMEM, 11885092, Thermo Fisher Scientific, Massachusetts, USA). The medium contained 1 g/L glucose and 25 mM HEPES, supplemented with 10% fetal bovine serum (FBS, 10270-106, Thermo Fisher Scientific, Massachusetts, USA), 1% antibiotic-antimycotic (AA, 161-23181, Fujifilm Wako Pure Chemical Corporation, Osaka, Japan), and 1% non-essential amino acids (NEAA, 11140-050, Thermo Fisher Scientific, MA, USA). Cultures were maintained at 37 °C in a humidified incubator with 5% CO_2_. The culture medium was replaced every two days throughout the expansion and experimental culture periods to maintain nutrient balance and cell viability.

In the FSSA-D configuration, the FSS group was cultured using dynamic perfusion within the FSSA placed on the cell desk. The control group was cultured on cell desks in standard well plates without the FSSA under conventional static conditions. This configuration allowed for a direct comparison between conventional static culture and the effect of applied FSS generated by the FSSA-D. In the FSSA-I configuration, cells were cultured on the basal side of a microporous membrane of a cell culture insert (Transwell^®^, 3450, Corning, New York, USA) in both the control group and the FSS group. This configuration allowed access to the basal membrane surface for FSS exposure.

Two types of extracellular matrix (ECM) coatings were prepared. For FSSA-D, a Collagen Type I-P solution was prepared by mixing 1 mL of Collagen Type I-P (634-00663, Nitta Gelatin, Osaka, Japan) with 9 mL of ultrapure water, followed by the addition of 10 µL of hydrochloric acid to adjust the pH to approximately 3. The FSSA-D was submerged in this solution and incubated at 37 °C for 1 h to allow for uniform coating of the microchannel surfaces. For the FSSA-I, Matrigel (354230, Corning, New York, NY, USA) was used at a 1:25 dilution in a cold medium and applied solely to the membrane surface of the cell culture insert. The inserts were incubated at 37 °C for 1 h to ensure a consistent ECM coating.

In case of cell seeding for FSSA-D, Caco-2 cells were seeded directly into the coated channels at 2 × 10^5^ cells/cm^2^. Approximately 80 µL of the cell suspension was introduced into each channel, followed by a 5 h pre-culture period in a 12-well plate containing the surrounding culture medium. After sufficient cell attachment, the FSSA-D were transferred into the BioStellar™ Plate to initiate perfusion culture.

In case of cell seeding for FSSA-I, inverted seeding facilitated basal membrane culture. The cell culture inserts were placed upside down in a 12-well plate, and 2 × 10^5^ cells/cm^2^ of Caco-2 cells were seeded onto the basal membrane surface using a custom-fabricated PDMS cup-like holder to retain the seeding medium. Each insert received 100 µL of medium and was incubated inverted for 3 h for cell attachment. Afterwards, the inserts were returned to their upright position and pre-cultured in a standard 24-well plate for 1 day, then transferred into the BioStellar™ Plate system for perfusion culture.

### 2.8. TEER Measurement

For the FSSA-I experiments, epithelial barrier formation was assessed using a transepithelial electrical resistance (TEER) measurement system (Millicell ERS-2, MERS00002, Merck Millipore, MA, USA). Chopstick electrodes were positioned with one probe in the apical compartment and the other in the basal compartment. TEER values were calculated by subtracting the background resistance of a blank insert from the resistance measured in the cell-seeded insert. TEER measurements were performed before each medium change to monitor barrier development under consistent conditions. TEER measurements were not conducted for the FSSA-D configuration. The flat-bottom culture chamber does not include a microporous membrane or separate apical and basal compartments, which are necessary for precise electrode placement and reliable resistance measurement.

### 2.9. Fluorescence Staining and Imaging

To visualize cell morphology and tight junction formation, cultured cells were prepared for fluorescence staining. Cells were rinsed with PBS (+) (D8662-500mL, Sigma-Aldrich, MA, USA) and fixed using 4% paraformaldehyde (163-20145, Wako Pure Chemical Corporation, Osaka, Japan) for 15 min. After rinsing with PBS (-) (D1408-500mL, Sigma-Aldrich, MA, USA) the cells were permeabilized with 1% Triton X-100 (02081155, KISHIDA CHEMICAL, Osaka, Japan) for 10 min, followed by additional washes with PBS (-). Samples were blocked using 1% BSA/PBS for 1 h at room temperature.

Tight junctions were stained using a primary ZO-1 antibody (21773-1-AP, Proteintech, IL, USA) diluted 1:500 in 1% BSA/PBS and incubated overnight at 4 °C in the dark. On the next day, cells were washed and stained with a combined solution of DAPI (340-07971, DOJINDO, Kumamoto, Japan) (1:1000), Alexa Fluor 488 Phalloidin (PHDG1, Cytoskelton.inc., Colorado, USA) (14 µM), and Alexa 568 secondary antibody (A10042, Thermo Fisher Scientific, Massachusetts, USA) (1:1000), all diluted in 1% BSA/PBS. The staining solution was applied to the inserts and incubated for 1 h at room temperature. After the final washes with PBS (-), the samples were stored in PBS (-) and imaged using a confocal laser microscope at the Tokai University Imaging Center for Advanced Research.

### 2.10. Statistical Analysis

All data are presented as the mean ± standard deviation (SD) derived from a minimum of three independent experiments. Statistical analysis was conducted using Student’s t-test, with statistical significance determined at a threshold of * *p* < 0.05, ** *p* < 0.01, and *** *p* < 0.005.

## 3. Results

### 3.1. Flow Analysis in FSSAs

**Figure 2:**
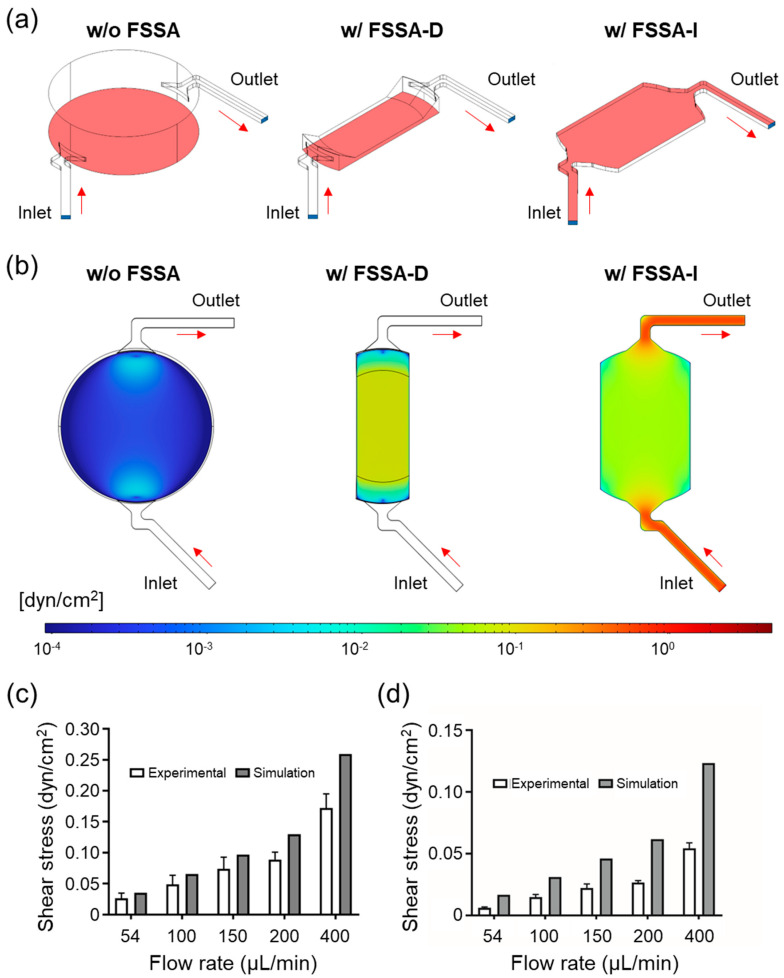
Flow simulation and experimental validation of the cell culture chamber in BioStellar™ Plate and fluid shear stress attachments (FSSAs). (**a**) Illustrations of the simulated cell culture chamber and mounted FSSAs, indicating inlet/outlet (blue) and analysis regions (pink): the bottom surface of the cell culture chamber for the BioStellar™ Plate, the channel floor for FSSA-D, and the flow region underneath the insert membrane for FSSA-I. (**b**) Simulated fluidic shear stress distributions at 100 µL/min in the XY-plane: the cell culture chamber exhibits low fluidic shear stress throughout the culture area, whereas the FSSA-D channel produces a stable, uniform shear profile, and the FSSA-I channel generates basal-directed laminar flow along the underneath of the insert membrane. (**c**) Comparison of simulated and experimentally measured shear stress for FSSA-D across various flow rates. (**d**) Comparison of simulated and experimentally measured shear stress for FSSA-I across various flow rates. Data are presented as mean ± SD from n = 3 (FSSA-D) and n = 3 (FSSA-I).

To evaluate the fluid dynamics within the BioStellar™ Plate and assess the performance of the developed FSSAs, we conducted a combination of finite element method (FEM) simulations and experimental flow validation. Figure 2a illustrates the simulated BioStellar™ Plate, highlighting the inlet and outlet flow paths, as well as the analysis region located at the bottom of the culture chamber. The open-top culture chamber format and internal geometry of the unmodified chamber limited control over directional flow. The XY-plane velocity cross-section showed highly non-uniform flow, with low-velocity zones at the center (Figure 2a,b, left), resulting in minimal shear exposure for centrally located cells (Figures S5 and S6, and Table S1).

To evaluate the functionality of the FSSAs, simulations were conducted under the same conditions as those of the BioStellar™ Plate. Simulation results demonstrated that both FSSA-D and FSSA-I effectively redirected the recirculating medium into well-controlled, parallel laminar flow regimes (Figure 2a, middle and right). For FSSA-D, the flow was guided through a narrow, straight channel (channel height: 0.4 mm), resulting in uniform, unidirectional laminar flow across the culture surface. The velocity profile confirmed even shear exposure across the entire width of the channel (Figure 2b, middle). Similarly, the FSSA-I attachment redirected the flow beneath the insert membrane, generating consistent laminar flow along the basal surface through a 0.5 mm high channel (Figure 2b, right). These designs successfully converted native circulation into stable, physiologically relevant FSS profiles.

Experimental validation was conducted using fluorescent bead tracking to measure shear stress at various flow rates: 54, 100, 150, 200, and 400 µL/min. For the FSSA-D configuration, experimental and simulation values showed good agreement at flow rates of 54, 100, and 150 µL/min, with no significant differences observed. However, notable discrepancies emerged at 200 and 400 µL/min, where experimental values were substantially lower than simulated predictions.

In contrast, the FSSA-I configuration exhibited consistent discrepancies between experimental and simulation values across all tested flow rates (Figure 2c,d). These discrepancies stem from the inherent difference between idealized simulation conditions and real fabrication tolerances. The simulations assume perfect dimensional accuracy, while the experimental setup involves fabricated components with dimensional variations. The FSSAs were 3D-printed and assembled by hand, and the BioStellar™ Plate itself does not possess perfectly precise dimensions. The different discrepancy patterns between FSSA-D and FSSA-I likely reflect their distinct sensitivities to dimensional imperfections. FSSA-D’s stagnation region design appears more tolerant to minor dimensional variations at lower flow rates, with fabrication effects becoming prominent only at higher velocities. FSSA-I’s geometry may be inherently more sensitive to dimensional deviations, resulting in consistent flow pattern alterations across all tested flow rates.

From the validated flow data, corresponding FSS values were calculated. In the FSSA-D system, a rotation speed of 5500 rpm generated an approximate flow rate of 89 µL/min, yielding a calculated FSS of 0.02 dyn/cm^2^ (Figure 2c). For FSSA-I, a rotation speed of 6500 rpm produced approximately 104 µL/min, resulting in a comparable FSS of 0.02 dyn/cm^2^ (Figure 2d). These conditions were selected based on prior studies indicating that low-level FSS in this range supports epithelial differentiation, barrier formation, and polarization in Caco-2 cells.

The findings indicate that FSSA-D and FSSA-I enabled the BioStellar™ Plate to generate stable and adjustable FSS environments. This system provides a platform for investigating FSS-dependent cellular responses under physiologically relevant flow conditions.

### 3.2. Cell Culture Evaluation in the FSSA-D

Following the characterization of fluid dynamics, the biological impact of FSS was evaluated using the FSSA-D configuration. Two conditions were implemented: a static control (cells cultured on the cell desk and placed directly in the well plate without flow), and a physiologically relevant low-FSS condition (5500 rpm; 89 µL/min; 0.02 dyn/cm^2^). This comparison enabled the assessment of shear stimulation effects while ensuring comparable cell viability between groups. Caco-2 cells were pre-cultured in FSSA-D for 5 h in standard 12-well plates before being transferred into the BioStellar™ Plate for dynamic culture over five days.

**Figure 3:**
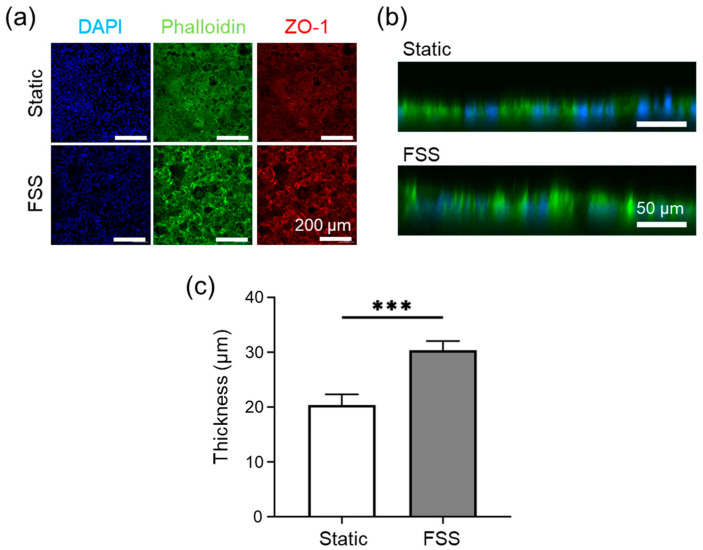
Caco-2 cells cultured under static and fluidic shear stress (FSS) conditions using FSSA-D. (**a**) Representative fluorescence images after 5 days of culture, stained for nuclei (DAPI, blue), F-actin (Phalloidin, green), and tight junctions (ZO-1, red). (**b**) Confocal cross-sectional images showing epithelial thickness under static and FSS conditions. (**c**) Quantitative comparison of epithelial thickness between static and FSS conditions. Data are presented as mean ± SD from n = 3 (static) and n = 3 (FSS); *** *p* < 0.005.

Fluorescence staining with DAPI (nuclei), Phalloidin (F-actin), and ZO-1 (tight junction protein) was performed on day 5 to evaluate cellular morphology, cytoskeletal arrangement, and junctional organization. Confocal images confirmed that both static and FSS conditions supported the formation of confluent Caco-2 monolayers with well-developed cytoskeletal structures and continuous ZO-1 expression patterns (Figure 3a). No detachment or major morphological abnormalities were observed in either group, indicating that both conditions enabled stable short-term culture. A slightly enhanced continuity of ZO-1 signal was noted under FSS conditions, potentially reflecting FSS-mediated support for tight junction maturation, consistent with previously reported mechanosensitive pathways [15,16,17], though these differences remained subtle and within biological variation.

Vertical cross-sections of DAPI and Phalloidin-stained samples revealed further distinctions in epithelial architecture (Figure 3b). Cells exposed to FSS developed thicker, columnar morphologies with well-defined apical–basal polarity, while static conditions yielded shorter and less-polarized cell layers. Quantitative analysis showed a significant increase in cell thickness under FSS, with averages rising from 20 µm (static) to 30 µm (FSS) (Figure 3c).

### 3.3. Cell Culture Evaluation in the FSSA-I

**Figure 4:**
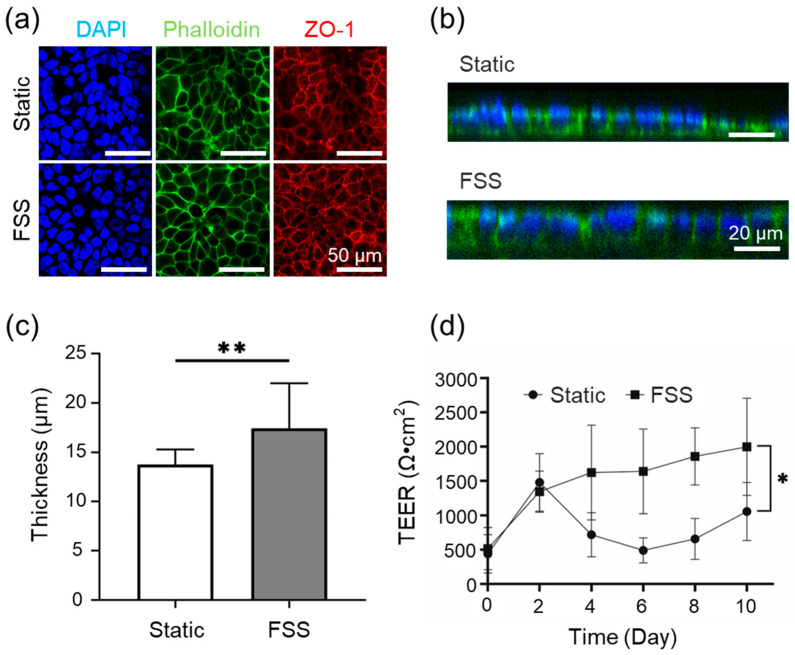
Caco-2 cells cultured under static and fluidic shear stress (FSS) conditions using FSSA-I. (**a**) Representative fluorescence images after 10 days of culture, stained for nuclei (DAPI, blue), F-actin (Phalloidin, green), and tight junctions (ZO-1, red). (**b**) Representative confocal z-stack images showing cross-sectional views of epithelial thickness. (**c**) Quantitative comparison of epithelial thickness between static and FSS conditions. Data are presented as mean ± SD from n = 15 (static) and n = 14 (FSS). (**d**) TEER measurements during 10 days of culture under static and FSS conditions. Data are presented as mean ± SD from n = 6 (static) and n = 6 (FSS); * *p* < 0.05; ** *p* < 0.01.

The biological response of epithelial cells under controlled FSS was evaluated using the FSSA-I configuration. Dynamic perfusion at 6500 rpm with the BioStellar™ Plate system produced a flow rate of approximately 104 µL/min, corresponding to an estimated 0.02 dyn/cm^2^ FSS. Caco-2 cells were cultured under static and FSS conditions for 10 days, with fluorescence staining evaluating morphology and junctional organization. Confocal images stained for DAPI, Phalloidin, and ZO-1 confirmed confluent monolayers in both groups, and well-developed cytoskeletal architecture with continuous ZO-1 patterns; no detachment or structural abnormalities were evident, indicating support for viable long-term culture (Figure 4a). Vertical cross-sectional imaging of DAPI and Phalloidin-stained samples showed that, under FSS conditions, cells displayed greater apical–basal polarity and increased vertical height than static controls (Figure 4b and Figure S7). Quantitative analysis showed a statistically significant increase in epithelial thickness under FSS (*p* < 0.01): from ~14 µm (static) to ~17 µm (flow) (Figure 4c). Despite comparable morphology, TEER (transepithelial electrical resistance) measurements over 10 days revealed functional differences in barrier integrity, whereby static conditions led to inconsistent TEER after day 2, while FSS induced a steady increase, stabilizing at higher levels (Figure 4d).

## 4. Discussion

In this study, we developed and evaluated two FSSAs, FSSA-D and FSSA-I, for integration with the BioStellar™ Plate microphysiological system platform. These attachments enable the precise application of low-level FSS to epithelial cell cultures. Our combination of computational simulations and experimental flow validation demonstrated that both attachments effectively transformed the previously irregular recirculating flow within the BioStellar™ Plate chamber into stable, uniform laminar flow environments [18]. The controlled hydrodynamic conditions produced by these devices facilitated reproducible FSS loading.

Our modular FSSA design can be adapted to a range of commercial MPS. By converting the open-top culture chamber’s recirculating flow to a controlled laminar flow, this approach is applicable to MPS platforms such as CN Bio’s PhysioMimix (CN Bio, Cambridge, England) and other open-top culture chamber-format MPSs [19]. Our work introduces a design concept that can be adapted for use with other commercial MPSs that share similar open-top culture chamber formats. Although we developed our attachments for the BioStellar™ Plate’s standard microplate size, the core design principles and modular approach offer a flexible framework that can be adjusted for other MPS platforms. While we validated the design with intestinal epithelial cells, the controlled FSS environment created by these attachments could also benefit other mechanosensitive cell types. For example, endothelial cells, essential for vascular modeling and dependent on physiological fluidic shear stress for proper function, could benefit [13,16]. Offering controlled mechanical stimulation without changing standard culture formats, these accessories can advance multi-organ and disease modeling research across the MPS field.

Biological assessments utilizing Caco-2 cells indicated that static and FSS conditions using both FSSA-D and FSSA-I supported the formation of a confluent monolayer. Cells exposed to controlled FSS exhibited enhanced transepithelial electrical resistance (TEER), increased epithelial layer thickness, and improved barrier function compared to static controls. The FSS of 0.02 dyn/cm^2^ in the cell culture experiment is a physiologically relevant mechanical stimulus closely mimicking the in vivo environment for intestinal epithelial cells [20,21,22,23]. This value has been reported as optimal in prior studies: for example, Shin et al. demonstrated that flow-dependent physical cues control human intestinal morphogenesis in microengineered gut-on-chip systems, while other research has shown that physiological shear stress enhances differentiation, mucus formation, barrier integrity, and 3D tissue architecture at this stress level [20,21,22,23,24,25]. Most notably, quantitative functional assessment of epithelial barrier properties, as measured by TEER, was possible in the FSSA-I configuration [26]. Here, the application of FSS resulted in a progressive increase and stabilization of TEER values, indicative of tighter junctions and improved barrier integrity [27,28]. These results corroborate the extensive literature indicating that mechanical cues from physiologically relevant FSS accelerate epithelial maturation, enhance cytoskeletal and junctional organization, and support the development of more predictive and robust in vitro intestinal models [20,21,22,23,29]. These findings confirm that low-level FSS improves architecture and barrier function, demonstrating the effectiveness of the BioStellar™ Plate system with FSSAs and its reliability in modeling physiological epithelial responses [25,30,31].

In conclusion, incorporating these FSSAs significantly enhances the functional capabilities of the BioStellar™ Plate platform by introducing controllable mechanical stimulation while ensuring compatibility with standard culture formats. These design principles lay the groundwork for using controlled fluidic shear stress with different cell types and commercial MPS platforms. This could help more researchers use mechanical stimulation that better reflects in vivo conditions. Future studies that include a wider range of cell types, such as endothelial and hepatic cells, will help demonstrate the broad applicability of this approach for disease modeling and drug development [8]. Our FSSAs provide a straightforward and accessible approach for mimicking physiologically relevant flow conditions in vitro. Our attachments help investigate FSS-mediated effects on epithelial cell function and intestinal barrier models within MPSs.

## 5. Future Directions and Applications

The FSSAs have overcome the limitations of current MPSs by offering a modular on-chip method for applying fluidic shear stress. The FSSAs enable more accessible implementation than conventional systems that require complex external equipment, such as commercial platforms with peristaltic pumps or pneumatic pumps, necessitating specialized tubing networks and pressure control systems [32]. The advancement responds to the increasing demand for simplified, cost-effective alternatives to open-top culture chambers designed for MPS platforms with an integrated pump. The open-top culture chambers designed for MPS platforms with integrated pumps, such as BioStellar™ Plate, feature open-top cell culture chambers that allow for easy handling of cell maintenance and are capable of circular flow between two cell culture chambers. However, the open-top culture chamber designed has the disadvantage of a larger dead volume in the culture medium and has difficulty applying fluidic shear stress, which frequently hinders the widespread adoption of open-top culture chambers designed for MPS platforms [2].

Our research’s modular design supports integration with existing workflows and accommodates a variety of tissues that are mechanosensitive beyond the intestinal epithelium. Future work will expand the range of fluidic shear stress and validate performance across multiple cell types and tissue models. FSSAs’ potential for standardization across research applications makes them a significant contribution to the development of MPSs, especially for laboratories aiming to introduce controlled mechanobiological stimulation without extensive infrastructure. 

## Data Availability

The original contributions presented in this study are included in the article/Supplementary Materials. Further inquiries can be directed to the corresponding author.

## Supplementary Materials

The following supporting information can be downloaded at: https://www.mdpi.com/article/10.3390/mi16091051/s1, Figure S1: Design and dimensions of the fluidic shear stress attachments; Figure S2: The custom-fabricated PDMS-based cell culture chamber model of the BioStellar™ Plate; Figure S3: Structural diagrams and flow visualization of fluidic shear stress attachments; Figure S4: Comparison of average and maximum flow velocities at various flow rates for FSSA-D (upper) and FSSA-I (lower) configurations; Figure S5: Simulated fluidic shear stress distribution at the bottom of the BioStellar™ culture chamber; Figure S6: Quantitative analysis of fluidic shear stress at the bottom of the cell culture chamber in the BioStellar™ culture chamber; Figure S7: Confocal images of FSSA-I show epithelial morphology under static and FSS conditions; Table S1: Average and maximum fluidic shear stress values at the bottom of the cell culture chamber in the BioStellar™ Plate; Movie S1: Dynamic visualization of fluid flow in the FSSA-D configuration using fluorescent microbeads; Movie S2: Dynamic visualization of fluid flow in the FSSA-I configuration using fluorescent microbeads.
