# Supplementary material for: The Fluidic Shear Stress Loading Method Enables Mechanobiological Stimulation in an On-Chip Pump-Integrated Microphysiological System
Source: Micromachines (Basel). 2025 Sep 15;16(9):1051. doi: 10.3390/mi16091051 (PMC12471289; doi:10.3390/mi16091051)
Supplement: Supplementary file 1 [file micromachines-16-01051-s001.zip › micromachines-3836464-supplementary-send conversion-done.pdf]

micromachines

Supplementary Materials: The Fluidic Shear Stress Loading Method Enables Mechanobiological Stimulation in an On-Chip Pump-Integrated Microphysiological System

Jin Hong Yap [1], Satoshi Ishizaki [1], Hiroko Nakamura [2], Kenta Shinha [2] and Hiroshi Kimura [1,2,*]

[1] Department of Mechanical Engineering, School of Engineering, Tokai University, 4-1-1 Kitakaname, Hiratsuka 259-1292, Japan
[2] Micro/Nano Technology Center, Tokai University, 4-1-1 Kitakaname, Hiratsuka 259-1292, Japan
* Correspondence: hkimura@tokai.ac.jp

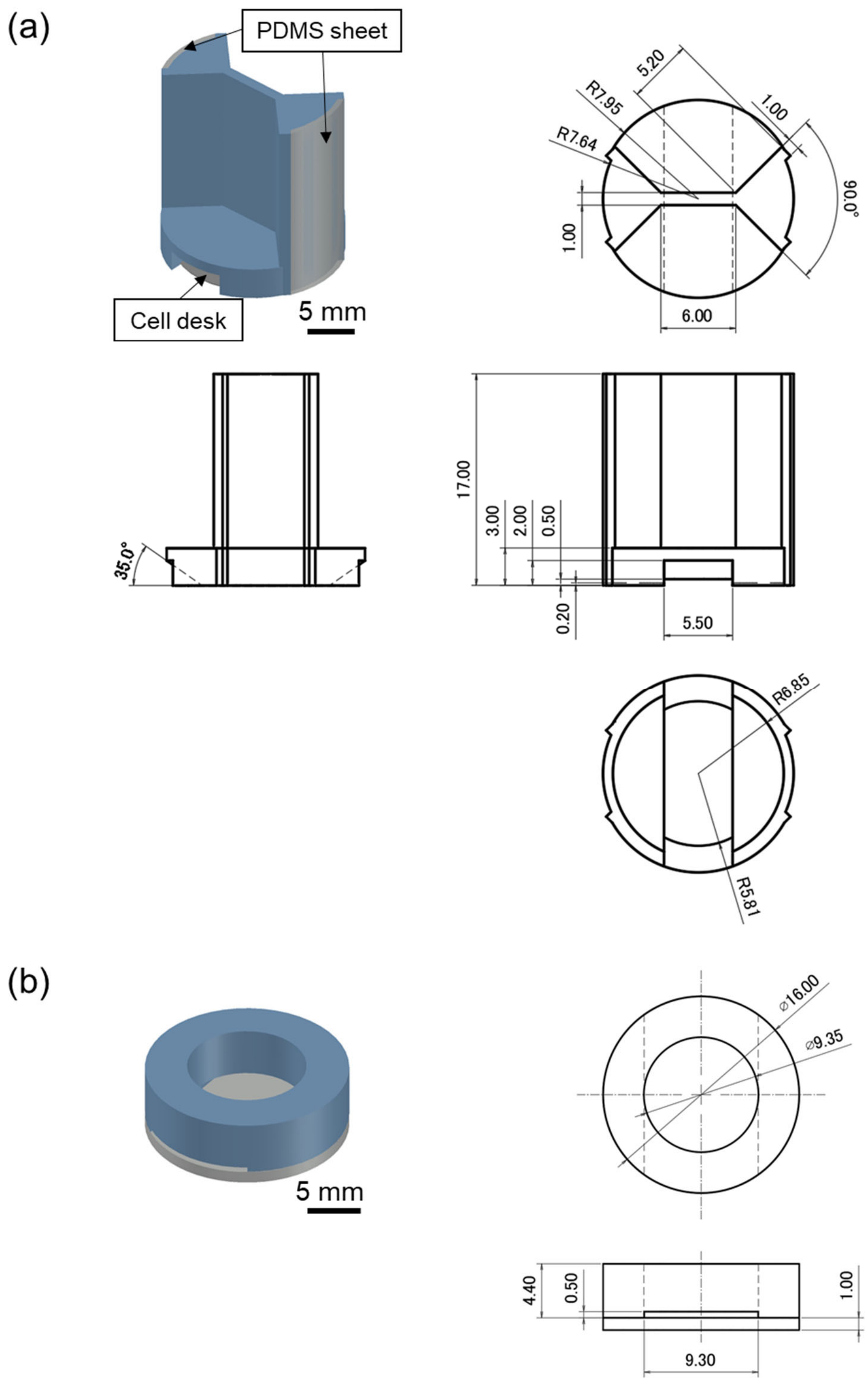

Figure S1. Design and dimensions of the fluidic shear stress attachments. (a) CAD rendering and detailed dimensions of the fluidic shear stress attachment (FSSA-D), showing the integrated cell desk and the PDMS sealing sheet. (b) CAD rendering and detailed dimensions of the cell culture insert-based fluidic shear stress attachment (FSSA-I), designed to accommodate standard cell culture inserts.

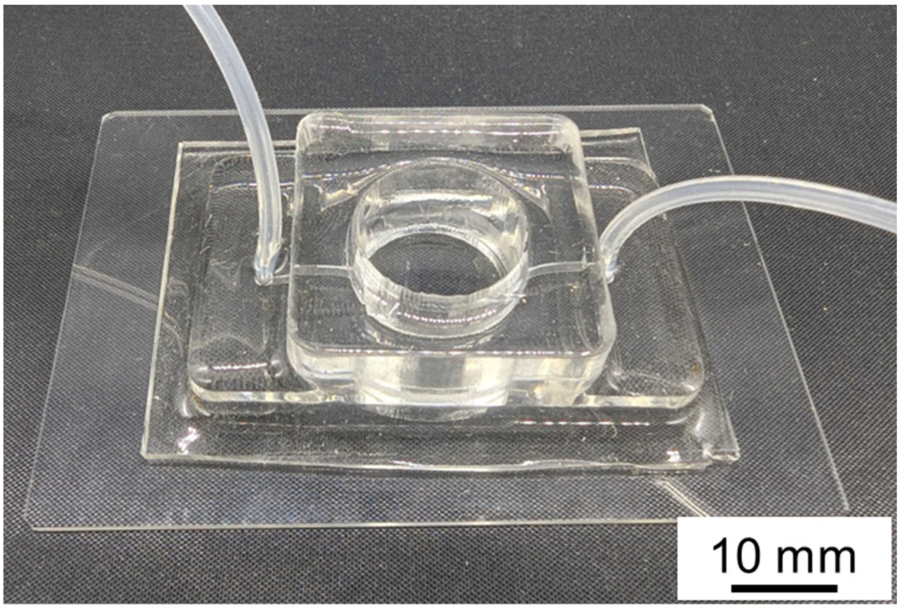

Figure S2. The custom-fabricated PDMS-based cell culture chamber model of the BioStellar™ Plate. The model was designed for flow observation experiments, featuring inlet and outlet ports that enabled controlled perfusion through the culture chamber area. The glass-bottom structure supports high-resolution imaging for direct visualization of fluid dynamics.

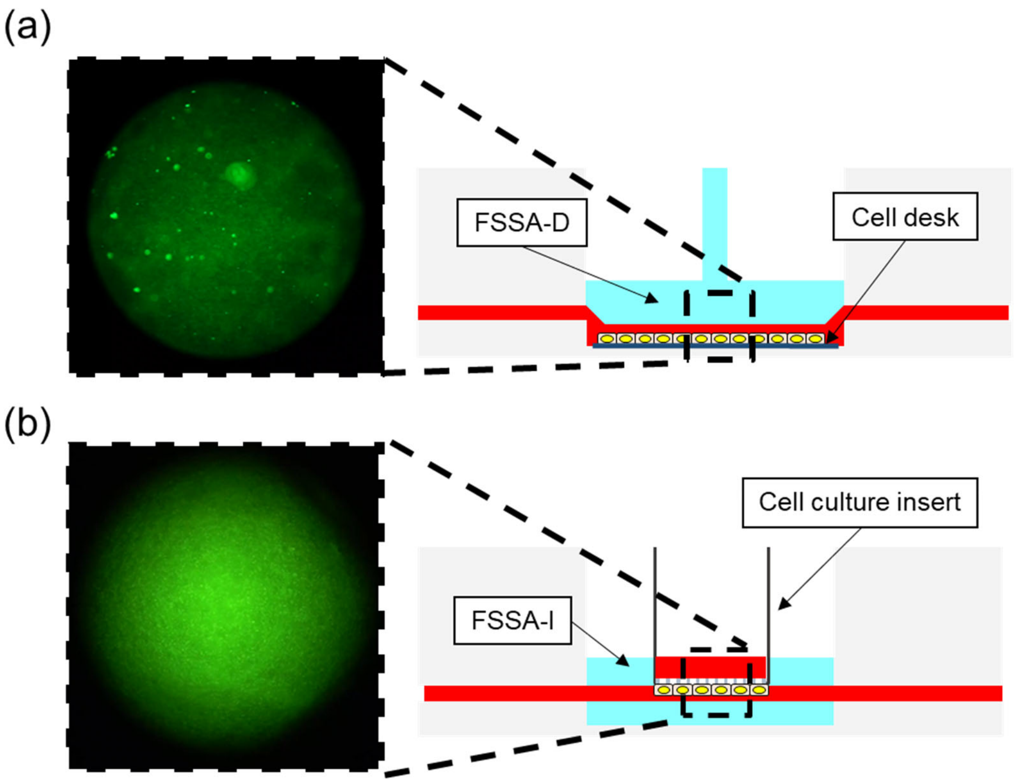

Figure S3. Structural diagrams and flow visualization of fluidic shear stress attachments. (a) Representative image of fluorescent microbeads captured in the FSSA-D configuration during flow validation experiments using the BioStellar™ plate. (b) Representative image of fluorescent microbeads captured in the FSSA-I configuration during flow validation experiments using the BioStellar™ plate.

Movie S1. Dynamic visualization of fluid flow in the FSSA-D configuration using fluorescent microbeads. The video demonstrates that the flow has been redirected to the cell desk cell culture area, resulting in a uniform flow that can maintain mechanobiological stimulation to the cell culture area on the cell desk.

Movie S2. Dynamic visualization of fluid flow in the FSSA-I configuration using fluorescent microbeads. The video shows the flow through the bottom of the cell culture insert's porous membrane, enabling continuous and uniform flow that maintains mechanobiological stimulation to the cell culture area.

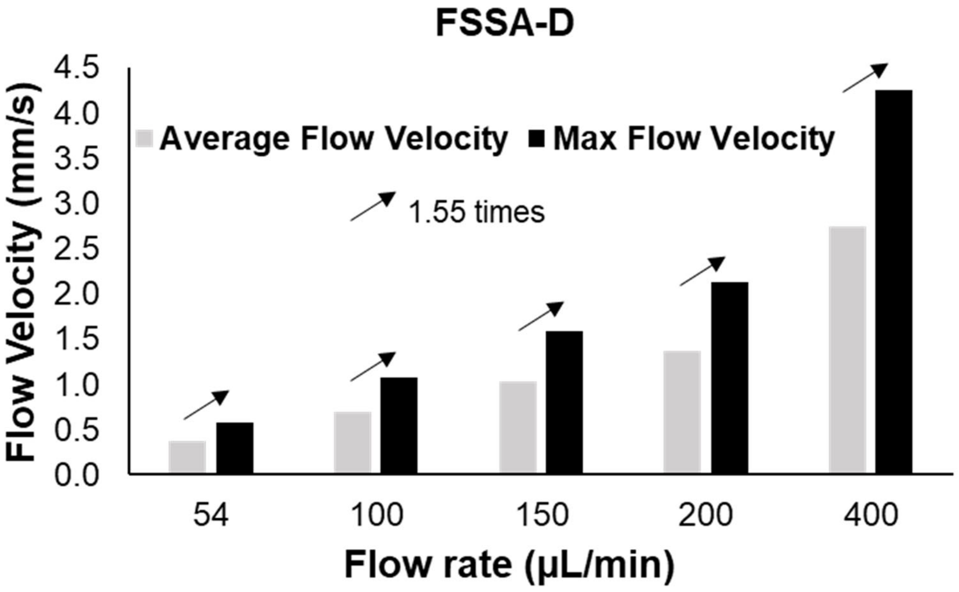

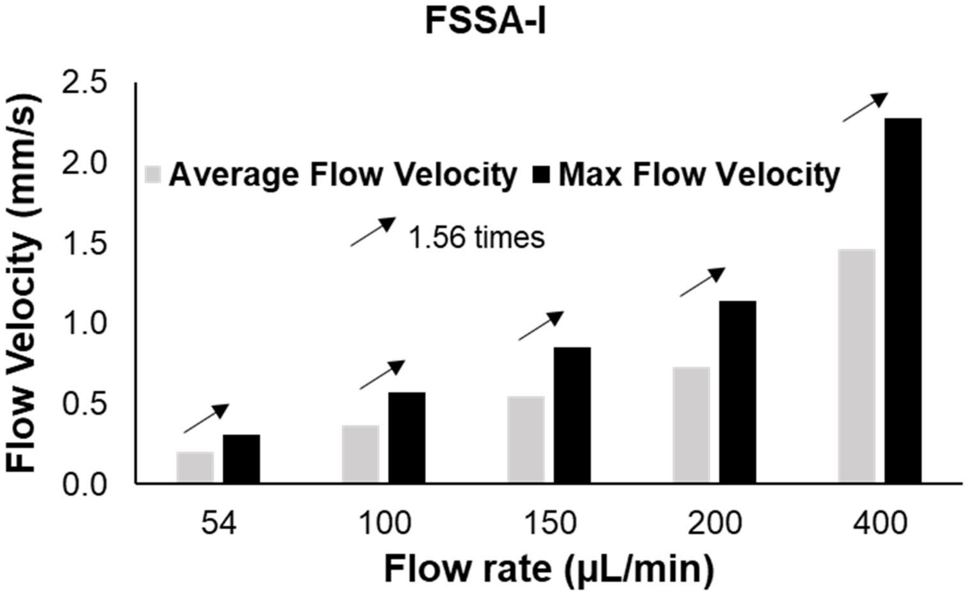

Figure S4. Comparison of average and maximum flow velocities at various flow rates for FSSA-D (upper) and FSSA-I (lower) configurations. Correction factors from ANSYS Fluent simulations (1.55 for FSSA-D, 1.56 for FSSA-I) were applied to convert peak velocities into average values due to the non-cylindrical channel geometry. This correction was critical for improving the accuracy of our fluidic shear stress loading estimations. By combining simulated flow profiles with experimental flow data, we could better define the actual fluidic shear stress environment experienced by the cultured cells, providing a solid basis for interpreting how fluidic shear stress influences epithelial morphology and barrier formation in our system.

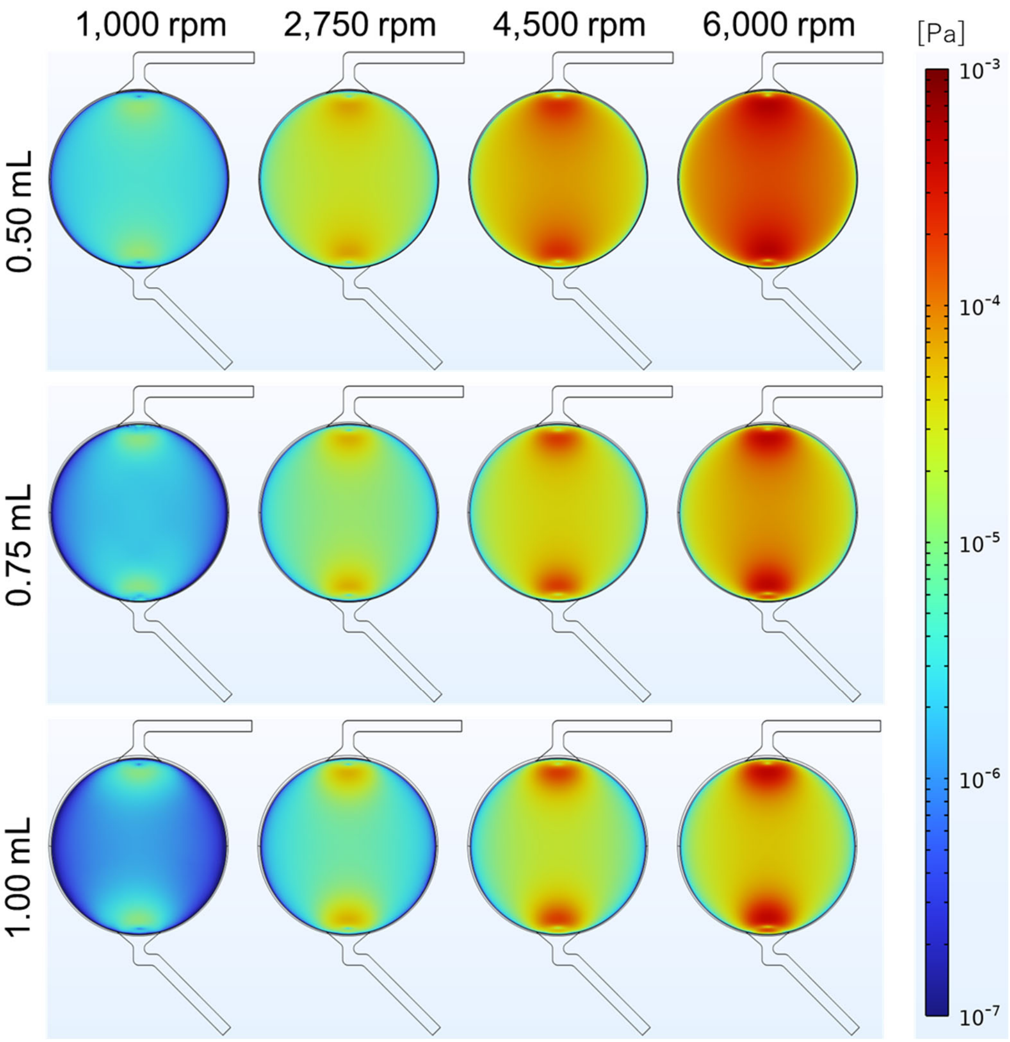

Figure S5. Simulated fluidic shear stress distribution at the bottom of the BioStellar™ culture chamber. Fluidic shear stress distribution is shown under varying chamber fill volumes and stirrer motor rotation speeds. Fluidic shear stress magnitude is in pascals (Pa).

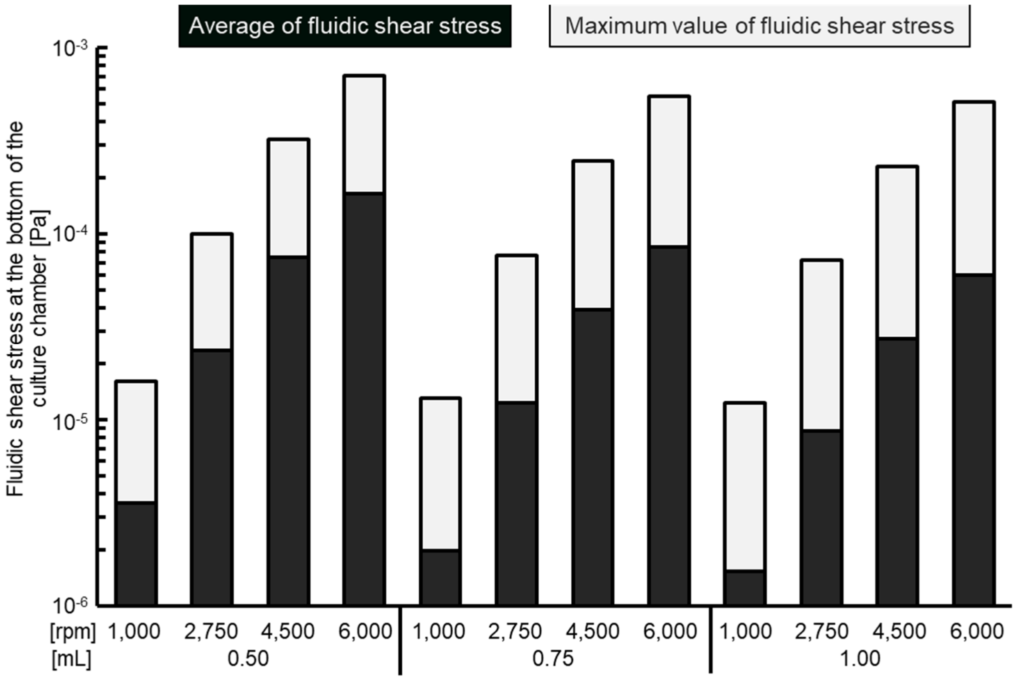

Figure S6. Quantitative analysis of fluidic shear stress at the bottom of the cell culture chamber in the BioStellar™ culture chamber. Data shows how fluidic shear stress magnitude increases with both rotational speed and chamber volume.

Table S1. Average and maximum fluidic shear stress values at the bottom of the cell culture chamber in the BioStellar™ Plate. Results show the increase in fluidic shear stress with higher rotation speeds and lower medium volumes.

10^{-6} Pa	0.50 mL				0.75 mL				1.00 mL			
	1,000 rpm	2,750 rpm	4,500 rpm	6,000 rpm	1,000 rpm	2,750 rpm	4,500 rpm	6,000 rpm	1,000 rpm	2,750 rpm	4,500 rpm	6,000 rpm
Ave.	3.6	23.6	75.1	164.1	2.0	12.3	39.1	85.3	1.5	8.6	27.3	60.0
Max.	12.5	76.6	245.3	546.6	11.1	64.7	208.3	463.0	10.8	63.6	202.8	450.0

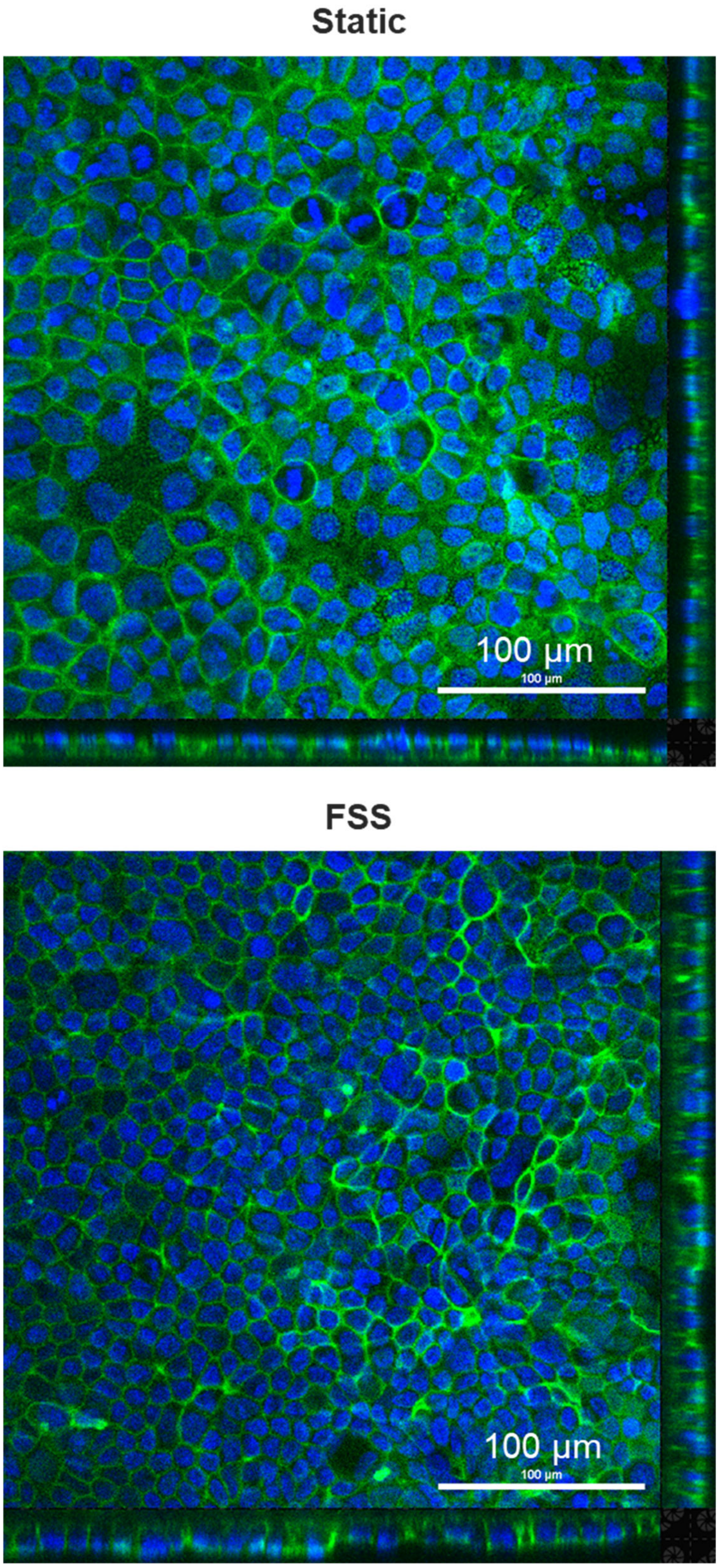

Figure S7. Confocal images of FSSA-I show epithelial morphology under static and FSS conditions. DAPI and Phalloidin staining reveal enhanced cell height and apical-basal organization under FSS conditions compared to static culture. Cross-sectional views are shown as side projections.
